# Progressive Oncological Surgery Is Associated with Increased Curative Resection Rates and Improved Survival in Metastatic Colorectal Cancer

**DOI:** 10.3390/cancers11020218

**Published:** 2019-02-14

**Authors:** Florian Primavesi, Stefan Stättner, Tarkan Jäger, Georg Göbel, Jaroslav Presl, Katerina Tomanová, Selina Buchner, Manuel Maglione, Thomas Resch, Jörg Hutter, Dietmar Öfner, Adam Dinnewitzer

**Affiliations:** 1 Department of Visceral, Transplant and Thoracic Surgery, Medical University of Innsbruck, 6020 Innsbruck, Austria; florian.primavesi@tirol-kliniken.at (F.P.); manuel.maglione@tirol-kliniken.at (M.M.); t.resch@tirol-kliniken.at (T.R.); dietmar.oefner@i-med.ac.at (D.Ö.); 2 Department of Surgery, Paracelsus Medical University, 5020 Salzburg, Austria; ta.jaeger@salk.at (T.J.); j.presl@salk.at (J.P.); catherine.toma@gmail.com (K.T.); s.buchner@salk.at (S.B.); j.hutter@salk.at (J.H.); a.dinnewitzer@salk.at (A.D.); 3 Department of Medical Statistics, Informatics, and Health Economics, Medical University of Innsbruck, 6020 Innsbruck, Austria; georg.goebel@i-med.ac.at

**Keywords:** colorectal cancer, metastases, surgery, liver resection, pulmonary resection, peritoneal surface surgery, curative intent, resectability, modern chemotherapy, advances in management

## Abstract

Background: Secondary resection rates in first-line chemotherapy trials for metastatic colorectal cancer (mCRC) remain below 15%, representing a clear contrast to reports by specialised surgical centres, where progressive liver, peritoneal-surface, and pulmonary surgery increased access to curative-intent treatment. We present a long-term evaluation of oncosurgical management in a single-centre, analysing the aggregate effect of gradual implementation of surgical subspecialties and systemic treatments on mCRC patients’ resection rates and prognosis. Methods: Patients with newly diagnosed mCRC from 2003 to 2014 were retrospectively categorised into palliative treatment (PAT) and curative intent surgery (CIS) and three time periods were analysed for treatment changes and factors associated with survival. Results: Four hundred-twenty patients were treated (PAT:250/CIS:170). Over time periods, the number of presenting patients remained consistent, whereas curative resection rates increased from 29% to 55%, facilitated by an increment of patients undergoing hepatectomy (21 to 35%), pulmonary surgery (6 to 17%), and peritonectomy/intraoperative chemotherapy (0 to 8%). Also, recently, significantly more multi-line systemic treatments were applied. The median survival markedly improved from 21.9 months (2003–2006; 95% confidence interval (CI) 17.3–26.5) to 36.5 months (2011–2014; 95% CI 26.6–46.4; *p* = 0.018). PAT was a significant factor of poor survival and diagnosis of mCRC in the latest time period was independently associated with a distinctly lower risk for palliative treatment (odds ratio 0.15). Conclusions: In modern eras of medical oncology, achieving appropriate resection rates through utilization of state-of-the-art oncological surgery by dedicated experts represents a cornerstone for long-term survival in mCRC.

## 1. Introduction

Colorectal cancer (CRC) is the second most common cause of cancer mortality and also ranks second place with a cancer incidence of 447,000 new cases per year in Europe [1]. More than 20% of patients present with synchronous metastases at first diagnosis (stage IV) [2], and further 25–30% develop metastases during the course of the disease. Epidemiologic studies show that the liver is most commonly affected (colorectal liver metastases (CRLM)), representing 15% of newly diagnosed CRC, followed by the lung (11%) and the peritoneum (5%) [3,4,5].

Due to clinical heterogeneity of these patients, optimal treatment is not well-defined, hence oncosurgical management is challenging since multiple factors need to be considered, such as resectability of the primary tumour and metastases, mutational status, presence of symptoms and patient comorbidities. Consequently, there is a wide variation in overall survival (OS). Without any anti-tumour therapy, median survival rates remain below 6 months [6]. With novel chemotherapy regimens (5-Fluorouracil (FU) and Leucovorin with oxaliplatin (FOLFOX) or with irinotecan (FOLFIRI)) including biologicals like anti-epidermal growth factor receptor (EGFR) or vascular endothelial growth factor (VEGF) antibodies, the median survival has risen up to 24 months [7] with 5-year OS rates ranging from 10 to 20% [8]. The benefit of complete resection of all metastatic disease has been established in numerous clinical trials with 5-year survival surpassing 50% following hepatectomy for synchronous CRLM [9,10]. However, less than one quarter of patients can be offered curative surgery [11,12] in most countries due to the extent of the disease or lack of experienced hepatobiliary (HB) surgeons [13]. Therefore, the majority of patients with metastatic CRC (mCRC) are still being managed with palliative intent. Comparable to CRLM, significant survival benefits could also be demonstrated with curative-intent resection of pulmonary metastases or utilization of cytoreductive surgery (CRS) and hyperthermic intraperitoneal chemotherapy (HIPEC) for peritoneal carcinomatosis.

Several prospective randomised and retrospective studies have shown superior survival for patients with advanced hepatic metastatic involvement treated sequentially with potent chemotherapeutic agents and surgery [8,9,14,15]. The phase 2 trial CELIM reported a response rate of around 60% after FOLFOX or FOLFIRI combined with cetuximab in initially unresectable CRLM [16]. In this study, the secondary resection rate was almost 50%, and the 5-year-OS in this subgroup reached 46%, comparable to primarily resectable cases [17]. Furthermore, it has been demonstrated, that determining the individual patient’s CRLM resectability by surgeons with expertise in hepatobiliary surgery leads to markedly higher resection rates [13], which also results in improved survival [18].

While there is an on-going trend to restrict surgical treatment of complex oncological patients to specialized, high-volume centres in many European countries such as the United Kingdom, Denmark, Norway or the Netherlands, no central regulation has been established in Austria by now. Furthermore, even though the 5-year OS for all stages of CRC has improved from 47% to 62% in the last 20 years in Austria [19], data for the subgroup of patients with metastatic disease are scarce. So far, only one national register study between 1998 and 2002 is available, reporting a 5-year OS of 10% for stage IV patients [20]. Hence, the effects of recent progressive developments in surgery and systemic therapy on real-life mCRC patient survival within the last decade remain indeterminate.

This retrospective study evaluates all patients with mCRC admitted to an Austrian high-volume university hospital, reporting an in-depth analysis of changes in metastatic patterns, resection rates, medical management and oncological outcomes over a time period of 12 years. With this clinical picture, we aim to determine the impact of adoption of progressive colorectal metastases surgery by specialised HB-, thoracic- and CRS/HIPEC-surgeons on survival in an increasingly multimodal treatment over the last decade.

## 2. Results

### 2.1. Patient Characteristics and Overall Survival

In total, 420 patients presenting with newly diagnosed mCRC were treated at our hospital from 2003 to 2014. One hundred and seventy (40%) at some time-point underwent curative-intent surgery (CIS) for their initial metastases along the course of the disease while 250 patients (60%) remained palliative receiving only palliative treatment (PAT). Patient characteristics with clinical and pathological data are depicted in Table 1 (CIS and PAT group) and in Table S1 (total cohort), and did not differ substantially, showing only minimal changes over the three study periods (e.g., distribution of initial metastases site).

Detailed clinical and tumour characteristics comparing CIS and PAT groups are illustrated in Table 2. As expected, in summary, PAT patients were significantly older and had higher American Society of Anaesthesiology (ASA) score than CIS patients. Also, their primary tumours were more often located in the right colon and more commonly had a higher or unknown tumour- and lymph-node stage as well as R-status. Metastases of PAT patients were significantly more often synchronous, with higher serum carcinoembryonic antigen (CEA) at diagnosis and more frequent simultaneous hepato-pulmonary, peritoneal and distant lymph-node involvement. PAT patients also more commonly received no or only 5-FU-based chemotherapy after diagnosis of metastatic disease.

**Figure 1:**
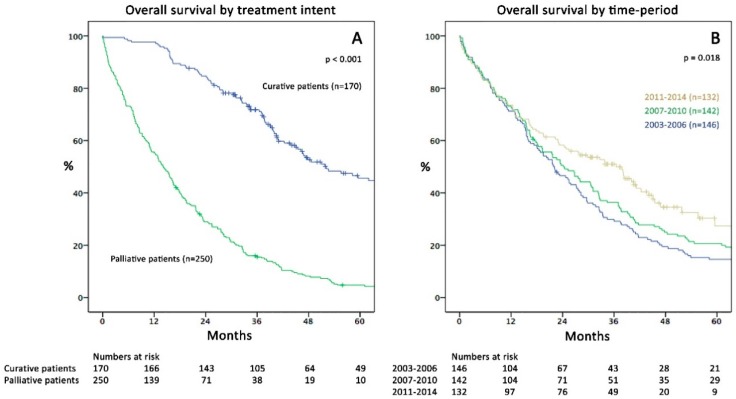
(**A**) Kaplan–Meier curves of overall survival (OS; months) comparing curative (CIS) and palliative (PAT) patients (*p* < 0.001). (**B**) Comparison of OS in the three study periods (*p* = 0.018).

The estimated median OS was 25.3 months (95%-CI 21.8 to 28.8) in the whole cohort, 52.1 months (95%-CI 39.9 to 64.3) in the CIS group, and 14.0 months (95%-CI 11.6 to 16.4) in the PAT group (*p* < 0.001; Figure 1A).

Analysing extent of metastatic involvement in the whole cohort, median OS for patients with liver-limited disease was 29.8 months (95%-CI 24.1–35.6) (*n* = 188; 45%), 46.8 months (95%-CI 34.7–60.0) for those with lung-limited disease (*n* = 57; 14%), 25.5 months (95%-CI 9.9–41.6) for liver-lung limited disease (*n* = 42; 10%) and 15.6 months (95%-CI 11.9–19.3) in case of any other organs involved (peritoneum, distant LN, etc.; *n* = 133; 32%; *p* < 0.001). In the CIS group, patients with liver-limited disease (*n* = 98; 58%) showed a median OS of 51.0 months (95%-CI 39.5–62.5) compared to 76.4 months (95%-CI 41.3–111.5) in cases with lung-limited disease (*n* = 39; 23%) and 39.2 months (95%-CI 34.8–43.6) in case of hepatopulmonary-limited metastatic disease (*n* = 11; 7%). Patients who had peritoneal metastases and underwent curative peritonectomy +/− HIPEC (*n* = 12; 7%) revealed a median OS of 55.6 months (95%-CI 22.6–88.7).

### 2.2. Survival and Surgical Data in the Three Time Periods

**Figure 2:**
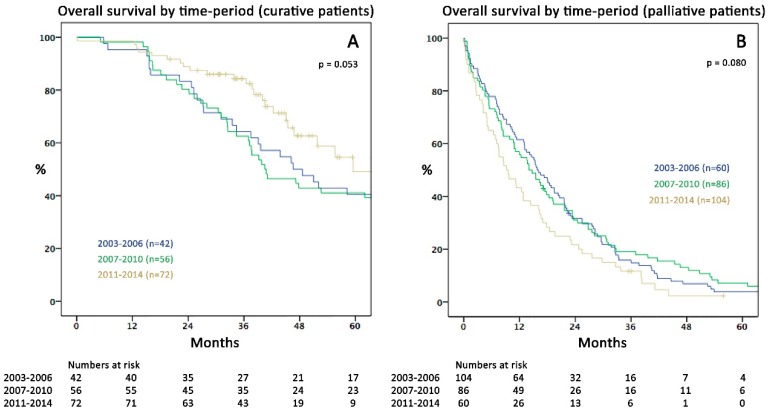
Kaplan–Meier curves of overall survival (months) comparing the three study periods in (**A**) curative (CIS; *p* = 0.053) and (**B**) palliative (PAT) patients (*p* = 0.080).

Over the three study periods, the median OS for the whole cohort increased from 21.9 months in 2003–2006 (95%-CI 17.3–26.5) to 36.5 months in 2011–2014 (95%-CI 26.6–46.4; *p* = 0.018; Figure 1B). Although there was a tendency towards better survival over time in the CIS group, this was not statistically significant (*p* = 0.053). Similarly, the recent time-trend of declining survival in the PAT subgroup was not significantly different to earlier periods (*p* = 0.080); Figure 2A,B).

**Figure 3:**
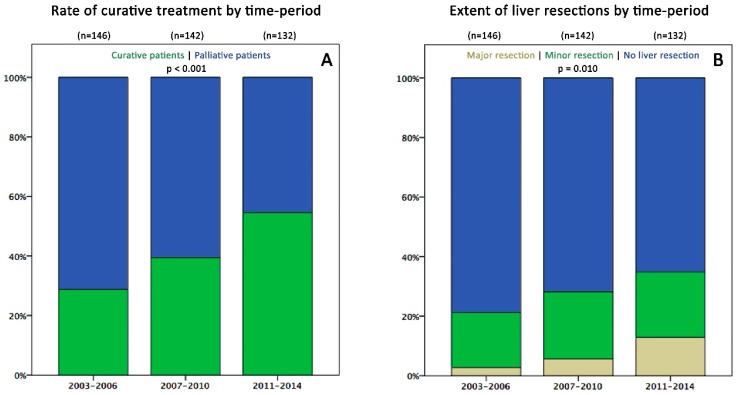
(**A**) Percentage of patients per period with curative-intent surgery (green) and palliative treatment (blue; *p* < 0.001). (**B**) Percentage of patients undergoing liver resection per period (blue = no liver resection; green = minor resection; beige = major resection (*p* = 0.010).

While the total number of patients per period was not significantly different (*n* = 146; 142; 134), the rate of CIS patients increased from 29% (*n* = 42) to 55% (*n* = 72) from 2003–2006 to 2011–2014 (*p* < 0.001, Figure 3A), due to an increase in liver resections from 21% to 35% (*p* = 0.041; Figure 3B), pulmonary resections from 6% to 17% (*p* = 0.013) and increased utilization of CRS and HIPEC (0% to 9%; *p* = 0.009) (Table 3). Hepatectomy patients showed a marked increase in frequency of bilobar metastases (19% to 52%; *p* = 0.004) and in the number of liver metastases per patient (mean: 2.2 to 5.5, median: 2 to 3; *p* = 0.005), with up to 40 lesions per patient in the last period, often treated by combined ablation and resection (CARE). This was accompanied by an increase of mild complications (Clavien-Dindo (C-D) 1-3a; 29% to 44%) but a concurrent decrease of severe complications (C-D 3b-4b; 16% to 4%; *p* = 0.024). Accordingly, this did not result in a prolonged length of stay (mean: 12.4 days lately) or a significant rise in mortality (0% to 2%; *p* = 0.460). Patients undergoing pulmonary resections recently had no severe morbidity and mortality although the rate of bilateral metastases increased from 0% to 22% (Table 3).

### 2.3. Disease Recurrence

In the CIS group, 128 patients (75%) relapsed after a median of 10.9 months (95%-CI 8.4 to 13.4) during follow up, showing a 5-year OS of 32% (median OS: 42.3 months; 95%-CI 36.4 to 48.2) compared to a 5-year OS of 85% in patients without recurrence (median not reached; *p* < 0.001). Sixty-three patients (49%) with relapse underwent further re-resections with curative intent (mean number of 1.6 treatments; range 1–5) resulting in a 5-year OS of 45% (median: 52.1 months; 95%-CI 42.3 to 61.9) compared to a 5-year OS of 18% in those consecutively treated with palliative therapy (median: 33.5 months; 95%-CI 29.5 to 37.5; *p* < 0.001). The recurrence rate after 3 years was not significantly different between 2003–2006, 2007–2010 and 2011–2014 (31%/20%/27%) with a median time to recurrence (TTR) of 10.7, 9.8 and 11.6 months, respectively (95%-CI 5.8 to 15.7, 5.1 to 14.5 and 8.5 to 14.8; *p* = 0.660).

### 2.4. Chemotherapeutic Regimens

Patients who received chemotherapy at any time after diagnosis of metastases (*n* = 341; 82%) showed a significantly better median OS of 29.8 months (95%-CI 25.7 to 34.0) compared to 4.0 months (95%-CI 2.7 to 5.3) in patients receiving no chemotherapy (*p* < 0.001). Reasons for omission of chemotherapy were comorbidities, limited performance status or individual patients’ decision to refuse chemotherapy. Evaluation of chemotherapeutics in the CIS group showed no significant differences within the whole study period (Table 3), except that no patient underwent 3rd/4th line agent therapy in the first study periods, which increased to 15% (*n* = 11; *p* = 0.007) in the 3rd time period, due to subsequent drug approval over the last years. Eighty-nine % of CIS patients (*n* = 151) received any type of chemotherapy after diagnosis of metastases, 38% (*n* = 64) underwent preoperative chemotherapy and 80% (*n* = 136) received postoperative chemotherapy after metastasectomy. Interestingly, the rate of CIS patients receiving dual (FOLFOX/FOLFIRI alone or sequentially) or triple chemotherapy (FOLFOXIRI) and the rate of regimens including biologicals also did not significantly change during the whole study period. Detailed data concerning chemotherapy for the whole cohort and palliative patients separately are displayed in the Table S2. In summary, there was no significant difference over time regarding the overall number of patients receiving any chemotherapy neither in the whole cohort nor in sub-analysis of the PAT group. However, recently a shift to more 5-FU-mono-based therapy (+/− biologicals) in the PAT group and an increased application of 3rd and 4th line agents in the whole cohort was recorded.

### 2.5. Factors Associated with Survival and Treatment Strategy:

Factors associated with OS in the whole cohort are presented in Table 4. In multivariable analysis, palliative treatment was the strongest factor independently associated with poor OS (Hazard ratio (HR) 3.68; 95%-CI 2.64–5.12; *p* < 0.001). The recent time period was significantly associated with improved OS (HR 0.67; 95%-CI 0.51–0.89) in univariable analysis. The HR of palliative treatment in the recent time period stratum (HR 8.99; 95%-CI 4.29–18.87) significantly differed from the first (HR 3.45; 95%-CI 1.88–6.36) and second time period (HR 2.65; 95%-CI 1.43–4.93). The multivariable Cox-regression model also showed, that high ASA-score was strongly associated with poor OS (HR: 3.50), as was mucinous/signet-cell primary tumour (HR: 3.18), receiving no chemotherapy or only 5-FU-based CTX compared to OX/IRI-based-regimens (HR: 2.57), CEA >200 ng/mL at diagnosis of metastases (HR 1.74), lymph node positive (HR: 1.52) and right-sided primary tumours (HR: 1.33).

Results of multivariable logistic regression for factors associated with palliative treatment decision are displayed in Table 5. Independently of patient and tumour characteristics, patients diagnosed with metastatic disease in the two recent time periods had a significantly lower risk of palliative treatment with an OR of 0.15 (2011–2014) and 0.54 (2007–2010), respectively. Moreover, metastatic disease exceeding isolated hepatic or pulmonary involvement (OR 6.73), synchronous disease (OR 2.43) and age ≥70 (OR 2.22) were significant factors leading to palliative treatment.

All patient data used for this study are provided in anonymized form in a supplement excel file (Table S3).

## 3. Discussion

Management and prognosis of mCRC patients have substantially changed over the last two decades. The present publication, to our knowledge, represents the first study by a European centre to portray these developments from a real-life perspective outside randomized trials. In the late 1990s, when FU and leucovorin were the only therapeutic options and hepatectomy was merely performed in cases with solitary metastases, the median OS was around 8–12 months [8]. Development of new chemotherapeutics and biologicals markedly increased treatment options since. Likewise, hepatic, pulmonary and peritoneal-surface surgery, as well as perioperative management, have undergone a striking development accompanied by reduced postoperative mortality and morbidity [21,22]. Consequently, our department alongside many others underwent a complete redefinition of our understanding of resectability [23]. This has led to an incremental gain in survival through increased resection rates based on a fundament of consequent application of potent chemotherapeutics: median OS increased from 21.9 months to 36.5 months within one decade. Although the actual 5-year OS rate is not yet available for the last time period (2011–2014), Kaplan–Meier estimates show a rate of 27%, presumably surpassing the 20% level of 2007–2010 (Figure 1B). Compared to nationwide 5-year OS rates of 10% in Austria before 2003 [20], this illustrates a major step forward in mCRC treatment. Comparable datasets from national or institutional mCRC registries outside Austria reported highly variable 5-year OS rates ranging from 8–9% (UK 2002-2006 [24], Norway and Denmark 2006–2008 [25]), 14% (USA 2006–2012, SEER Database [8,26]), 17% (Australia [27]), and 32% (USA 2004–2006, Mayo Clinic & MD Anderson [8]).

Our study revealed, that palliative treatment remains an independent factor strongly associated with poor OS (HR 3.68) in multivariable analysis. Bearing in mind the clinical limitations by other factors such as ASA status, primary tumour biology, metastatic extent and applicability of effective chemotherapy, the present findings clearly underline the crucial importance of aiming for curative intent. Patients should not only be discussed in a multidisciplinary team meeting (MDT) early in the course of mCRC disease. Moreover, resectability needs to be constantly re-evaluated with adequate surgical input to maximize CIS rates during systemic treatment with conversion potential. While the time period of first metastatic diagnosis—although significantly associated with OS in univariable analysis—could not be included in the final multivariable model due to multi-collinearity with treatment intent and other factors (e.g., ASA score), it is noteworthy that the rate of CIS patients substantially increased from 29% to 55% over time (Figure 3A) and the HR of palliative treatment for OS significantly increased over time from 3.45 to 8.99.

In our opinion, several conclusions may be drawn interpreting these findings in the context with results of logistic regression, where diagnosis in the recent time period was an independent factor protecting from palliative treatment (OR 0.15). First, optimization of patient selection for curative intent has markedly influenced OS (Figure 1B) by including more patients possibly benefiting from resection while at the same time precluding those with poor prognosis from unnecessary surgery (Figure 2A,B). This has mainly been achieved by strictly presenting all mCRC patients to MDT by oncologists and surgeons since 2009. Furthermore, gradual employment of experienced hepatobiliary surgeons led to implementation of an increasingly progressive approach in liver surgery e.g., through use of parenchymal sparing resection techniques, combined intraoperative ablation and resection [28,29], and consequent re-resections for recurrence. Secondly, this gain in resection rates was not accompanied by increased postoperative mortality or severe morbidity, in line with previously reported data of others [30,31,32]. The possibility of pushing resection rates without necessarily increasing complications is an important message to physicians that are not working within specialised centres. Besides growing numbers of hepatic resections (35%), also lung resections (17%) and CRS +/− HIPEC (8%) were performed more frequently recently. Inclusion of cases with advanced metastatic burden in the CIS subgroup is exemplarily demonstrated by hepatectomy patients’ characteristics: in the late period, the rate of major hepatectomies was 37%, fifty-three % of cases had bilobar disease and the mean number of liver lesions was 5.5 (range: 1–40). In summary, almost 50% of patients presenting with CRLM recently were treated curatively compared to less than 30% in the early period—a clear effect of surgical specialisation. Similar resection rates (53%) were reported by a large single centre study from Memorial-Sloan-Kettering Cancer Center, New York in 2009 [9] surpassing rates of 20 to 30% reported in studies by other centres [8,11].

Likewise, the observed increase in lung resections from 6% to 17% was accompanied by an increment of the mean number of lesions per patient (2.3) and the rate of bilateral disease cases (22%). Video-assisted-thoracoscopic-surgery (VATS) is utilized since the early 1990’s in our department and has since increasingly been applied also for mCRC patients, currently performed in about 40%. Although others have reported even higher rates of thoracoscopic resections (52%) for CRC lung metastases [33], in their series the majority of patients (83%) had only one single metastases. Concerns about intraoperatively missed metastases remains a major issue in VATS especially in patients with multiple lesions, possibly restricting further extent of the thoracoscopic approach [34]. Our CIS patients with lung-limited disease (*n* = 39; 23%) showed a median OS of 76.4 months (95%-CI 41.3–111.5) with an estimated 5-year OS of 57%, which is comparable to other recent series reporting 5-year OS rates between 43% and 68% [30,35], underlining the clear benefit of consequent pulmonary resection.

Introduction of CRS including HIPEC represents the third progressive development of mCRC surgery. In summary 12 (7%) CIS patients were treated curatively for initial peritoneal metastases, with a median OS of 55.6 months and a 5-year recurrence rate of 67% at a median TTR of 5.4 months (95%-CI 3.6 to 7.2). Studies included in a systematic review reported a wide range of median OS rates between 3.7 and 62.7 months after CRS+HIPEC [36] depending on inclusion criteria and chemotherapy protocol. The extent of peritoneal spread (peritoneal carcinomatosis index/PCI) and amount of successful tumour clearance (completeness of cytoreduction score/CCS) were identified as crucial factors influencing OS in these studies.

Concerning adoption of chemotherapeutics, due to participation in a number of oncological studies, combination-therapy schemes were early adopted for our patients. This might be the reason why no significant changes within the last 12 years have been recorded in CIS patients concerning the general rate of chemotherapy applications (89% in all CIS patients), administration of OX/IRI-based regimens (84%) or biologicals (63%). However, following subsequent drug approval, 21% of all patients and 15% of CIS patients received 3rd or 4th line agents in the latest period, building a fundament for disease stabilization that might further increase survival rates in the near future. Within the whole cohort, the rate of patients receiving any type of chemotherapy (81%) was very high compared to other international reports (e.g., data from the Netherlands: 55% from 2000–2004 [6]), underlining a very progressive approach to consequent systemic therapy application by our local oncologists in this state-of-the art multidisciplinary setting.

Recurrence is common after curative surgery in mCRC patients with extensive tumour burden, resulting in a substantial decline in OS. A recent international multicentre analysis has reported relapse after hepatectomy for CRLM in 47% of patients at a median of 10 months, with 5-year-OS rates of 74% in cases without recurrence compared to 58% with recurrence [37]. However, this study almost solely included liver-limited disease patients, whereas in our study only 58% had liver-limited disease. Accordingly, substantially higher recurrence rates of 75% of all CIS patients at a median of 10.9 months occurred in our cohort. Interestingly, recurrence rates did not increase over time, despite operating more patients with extensive disease. Also, the impact of freedom of recurrence on survival was even greater in our cohort compared to the aforementioned study, with 5-year OS of 87% for patients without recurrence compared to 28% in cases with relapse. The management of mCRC recurrence has changed substantially from mostly palliative treatment to a more progressive approach with multi-line chemotherapy and re-resections [38,39] to chronify the disease in cases where cure is unlikely. Within the whole study period 49% of CIS patients with recurrence received at least one episode of further metastases resection, leading to a median OS of 52.1 months (95%CI: 42.3–61.9) compared to 33.5 months (95%CI: 29.5–37.5; *p* < 0.001) after subsequent palliative therapy only. Standardised follow-up with repeat MDT discussion for possible re-resection (or ablation) to “reset the clock” represents the mainstay for good long-term survival rates [40].

In light of our results, the maximum proportion of mCRC patients generally judged eligible for curative surgery of around 20% in previous studies [8] could be challenged. In summary, over 50% of cases were treated curatively within recent years in an environment of modern multimodal treatment. This also underlines intriguing differences in the surgical management of patients in “real-life” situations compared to prospective first-line chemotherapy trials for mCRC, which are mostly being conducted by medical oncologists. In the PEAK and FIRE-3 trials, although only patients with a good performance status were included, only 13–14% [41,42] underwent hepatic resections and previous studies showed even lower rates of 3.3% to 6% [8]. While the PEAK and FIRE-3 trials exhibited a median OS of 25 to 34 months (depending on the RAS mutational status and type of received biological), estimated 5-year OS rates remained below 20% (compare with Figure 1B). It has previously been suggested, that increasing liver resections from 6% to 20% could provide a similar OS benefit (HR 0.86) as the addition of irinotecan to 5-FU (HR 0.78) in the first-line setting [8]. Hence, low rates of liver resections might represent lost opportunities for patients to achieve long-term survival. According to the incidence presented in the Austrian cancer statistics [19] our hospital was treating merely around 3% of all nationwide newly diagnosed synchronous mCRC cases. This emphasises the potential on nationwide survival rates through continuing centralisation or if a similar progressive approach would be applied in more areas of the country.

Aiming to further challenge current resectability rates, analysis of factors leading to palliative treatment adds insights into the mechanisms of clinical decision-making. In addition to the early time period of metastatic diagnosis, other variables strongly associated with increased likelihood of palliative treatment included metastatic extent beyond isolated hepatic or pulmonary metastases (OR 6.73), synchronous metastases (OR 2.43) and age ≥70 (OR 2.22). Regarding patients with simultaneous hepatic and pulmonary metastases we have shown, that CIS cases (*n* = 11) do present favourable outcome with a median OS of 39.2 months compared to 13.6 months in hepatopulmonary PAT cases (*n* = 31; *p* = 0.007). Similar to previous reports, this subgroup of patients shows a clear survival benefit comparable or only little lower than in those with isolated hepatic metastases (median OS: 51 months), when resection is feasible. However, potential progress of disease in the time between hepatic and pulmonary surgery and consequent inability of curative pulmonary treatment represents a relevant limitation, leading to markedly decreased OS (5-year-OS: 15-30%) in the literature [43,44]. Moreover, in our cohort, patients who had peritoneal metastases and underwent curative peritonectomy +/− HIPEC (*n* = 12; 7%) achieved a median OS of 55.6 months (95%-CI 22.6–88.7). A recent extensive systematic review and meta-analysis of 76 studies including >10,000 patients concluded, that CRS + HIPEC offers a significant OS benefit compared to traditional systemic palliative treatment (pooled HR 2.67) [45]. Increasing utilization of HIPEC and other concepts such as the ALPPS procedure [46], 3D-navigated-stereotactic ablation [47,48] and liver-directed therapies including hepatic arterial chemotherapy [49], selective internal radiotherapy (SIRT) [50] or transarterial chemoembolisation (TACE) [51] might additionally improve curative treatment rates and potentially OS. Concerning elderly patients, only 6 cases (10%) of octogenarians (patients aged >80) were treated curatively in our cohort, possibly representing a specific subgroup with potential for further increase of curative resection rates. However, available studies on the safety of liver resections for CRLM in octogenarians report conflicting results with partly significantly increased morbidity and mortality, most certainly demonstrating the need for strict preoperative risk assessment, for example with cardiopulmonary-exercise testing (CPET) [52,53].

Limitations of the present work include the retrospective design, the shorter follow-up time of the last time period cohort, possible inhomogeneity due to pooling of synchronous and metachronous disease patients and a conceivable referral bias due to inclusion of cases with external primary tumour resection. However, in multivariable analysis synchronous vs. metachronous disease had no impact on OS. Selection bias seems rather improbable, as we have included all cases of newly diagnosed mCRC, even those only treated with best supportive care, distinguishing our study from comparable others [8], which might translate to better generalisability. Prospective national registries for mCRC patients with data on curative treatment rates in individual centres could help to further determine the nationwide impact of specialised surgery on survival in the future. Finally, analysis of mutational status (KRAS, NRAS, BRAF, etc.) has only recently been routinely implemented in mCRC treatment at our department and could therefore not be systematically investigated over time.

In summary, the present data clearly point out the role of specialised surgery in mCRC supported by consequent systemic therapy in a multidisciplinary, multimodal setting, where surgeons can be the key drivers for continuous expansion of resectability boundaries leading to prolonged survival and potential cure. Ultimately, these real-life data accompanied by results from many other studies strongly foster the role of specialisation and centralisation on a national as well as a European level.

## 4. Materials and Methods

All consecutive patients presenting to Paracelsus Medical University Salzburg from 1 January 2003 to 31 December 2014 with newly diagnosed mCRC (synchronous or metachronous hepatic, pulmonary, abdomino-peritoneal or distant lymph-node metastases) were retrospectively evaluated. Within this period, developments of progressive mCRC surgery have been gradually implemented in our unit: For example, in 2009 and 2012 specialised HB-surgeons with an emphasis on mCRC treatment (D.Ö., S.S.) were employed, and in 2012 HIPEC was introduced by two colorectal surgeons (T.J., A.D.). Accordingly, to account for subsequent changes in the surgical management of mCRC patients, the study period was equally divided into three subperiods (2003–2006, 2007–2010, 2011–2014). Patients with previous curative intent therapies for metastases prior to the first presentation were excluded, while resection of the primary tumour at other centres was not an exclusion criterion. The study protocol was approved by the local medical ethics committee “Ethikkommission des Landes Salzburg” (protocol-number 415-EP/73/629-2016), which waived the need for written informed consent. The study was conducted and the results reported in accordance with the STROBE statement-checklist [54]. 

Our prospectively maintained and auditable CRC patient’s database was retrospectively complemented with detailed data about metastatic cancer surgery and palliative treatment from the hospital’s medical records. The following parameters were collected: date and patient age at first diagnosis of metastases, sex, American ASA classification, body mass index (BMI), serum CEA at time of first diagnosis of metastases, primary tumour/metastases location, type and duration of all (radio-)chemotherapy regimens during the whole course of the disease, primary tumour TNM (tumour, node, metastases) staging, residual tumour status, histological subtype and tumour grading, distant metastases characteristics (organ involvement; number, distribution and size of metastases in curatively treated patients), type of metastases surgery, postoperative morbidity (C-D classification [55]) and in-hospital mortality for primary and metastases surgery, time to recurrence as well as OS. Synchronous disease was defined as diagnosis of metastatic lesions within 6 months of the primary tumour diagnosis. In liver surgery, major resection was defined as resection of ≥ 3 anatomical segments [56] or ≥ 6 non-anatomical resections and ablations [57]. External medical reports were collected individually when crucial data were missing. 

Treatment of metastases was classified along the course of the disease as curative (CIS) when initially diagnosed metastases were completely resected with or without preoperative chemotherapy and no visible lesions were left intraoperatively (in sano resection). Cases of advanced metastatic burden (e.g., bilobar hepatic disease or simultaneous hepato-pulmonary lesions) requiring two- or three-stage surgical procedures were only classified as CIS when the last stage was successfully completed. Patients receiving chemotherapy for initially unresectable or borderline-resectable disease without successful conversion were classified as palliative (PAT). All other cases (no curative intent, progression during preoperative/palliative chemotherapy, best supportive care) were also classified as PAT. Resectability of CRLM between 2003 and 2008 was determined by senior general surgeons and multidisciplinary case discussion. From 2009 on resectability was classified by trained and experienced HB surgeons (D.Ö. and S.S.) according to patient- and tumour-based criteria exemplarily reported by Mattar et al. [23] and all patients were routinely discussed in our MDT. Criteria for resectability of pulmonary metastases over the whole study period were: sufficient residual functional pulmonary capacity alongside possibility of technically complete resection defined by a trained thoracic surgeon (J.H.). CRS with HIPEC was introduced in 2012 (T.J., A.D.) and indication was a PCI < 20 and a CCS of 0–1 after CRS (CCS 0 = no visible tumour remains; CCS 1 = residual tumour deposits < 2.5 mm).

### 4.1. Follow-Up

Follow-up after curative surgery was conducted according to the Austrian national guidelines [58]: The minimum follow-up regime consisted of clinical evaluation and CEA-level determination every 3 months during the first 3 years and every 6 months thereafter as well as chest and abdominal Computed Tomography (CT) scan every 6 to 12 months, and colonoscopy 12 and 60 months after surgery. Further imaging such as Positron Emission Tomography-Computed Tomography (PET-CT) or Magnetic Resonance Imaging (MRI) was added by individual decision. During chemotherapy, evaluation of response was performed with CT every 2 or 3 months (cycles), depending on the intended therapeutic approach (curative or palliative). Individual clinical follow-up and survival data were systematically cross-checked with the Statistics Austria national death registry. The last date of follow-up was 31 December 2016.

OS was calculated from index metastatic diagnosis to the date of last follow-up or date of death, TTR was calculated from the time of initial complete metastatic tumour clearance by surgery to the first event of recurrence as defined by Punt et al. [59].

### 4.2. Statistical Analysis

Patient characteristics are presented as frequencies (%) or as means (SD). Differences between time periods were assessed using the χ² and Fisher’s Exact Test for categorical variables and one-way analysis of variance (ANOVA) for continuous variables. For comparisons between the groups of palliative and curative patients also appropriate contingency tests were used for categorical variables and the Mann-Whitney-U Test for continuous variables. Kaplan–Meier curves were applied to visualise survival of subgroups and survival distributions were compared using the log-rank test. OS and TTR times are presented as medians with 95% confidence intervals; 5-year-OS rates are based on Kaplan–Meier estimations.

A Cox proportional hazard regression-model was fitted to estimate associations and 95% confidence intervals of relevant variables (based on literature and clinical practice) with OS. Significant factors of univariable analysis were included in the multivariable Cox model after sensitivity analysis for (multi-)collinearity. To take into account the interaction between time periods and initial-treatment strategy, we stratified the model for time periods. Multivariable logistic regression including all clinically relevant variables was performed to identify factors leading to palliative treatment. A p-value less than 0.05 was considered as significant throughout the analysis. For all statistical calculations SPSS version 21.0 (IBM Corporation, Armonk, NY, USA) was used.

## 5. Conclusions

This study underlines how gradually increased utilization of progressive oncosurgical techniques by dedicated hepatobiliary, colorectal, and thoracic surgeons can lead to markedly improved survival in mCRC patients. Many units rapidly implemented state-of-the-art combination chemotherapeutics as a profound fundament in multimodal treatment within the last years. However, the survival benefits achieved by appropriate resection rates through involvement of trained surgeons with expertise in the field appear underestimated and should, therefore, represent a major focus in efforts for further outcome improvements in the oncological community.

## Figures and Tables

**Table 1:** Demographic, clinical and pathological characteristics of the CIS (curative intent surgery) and PAT (palliative therapy) subgroup during the three study periods.

Variable	Curative Group (CIS)				Palliative Group (PAT)			
	2003–2006 *n* = 42	2007–2010 *n* = 56	2011–2014 *n* = 72	*p* *	2003–2006 *n* = 104	2007–2010 *n* = 86	2011–2014 *n* = 60	*p* *
Age at mCRC dx: Mean (SD)	66.0 (9.8)	65.0 (10.6)	63.7 (10.7)	0.507 †	67.0 (11.5)	70.2 (11.6)	74.0 (11.2)	0.001 †
BMI: Mean (SD)	25.9 (4.1)	24.9 (3.5)	25.2 (4.5)	0.487 †	25.3 (4.2)	25.9 (4.7)	24.7 (4.7)	0.276 †
Sex: Male	32 (76%)	32 (57%)	44 (61%)	0.130	68 (65%)	52 (61%)	34 (57%)	0.524
ASA				0.519				0.144
I	3 (7%)	7 (13%)	5 (7%)		10 (10%)	8 (9%)	1 (2%)	
II	25 (60%)	34 (61%)	47 (65%)		46 (45%)	31 (36%)	23 (38%)	
III	14 (33%)	13 (23%)	20 (28%)		38 (37%)	42 (49%)	27 (45%)	
IV/V	0 (0%)	2 (4%)	0 (0%)		9 (9%)	5 (6%)	9 (15%)	
Primary TU Location				0.821				0.085
Colon	22 (52%)	30 (54%)	37 (51%)		64 (62%)	59 (69%)	47 (78%)	
Rectum	19 (45%)	26 (46%)	33 (49%)		40 (39%)	25 (29%)	12 (20%)	
Both	1 (2%)	0 (0%)	2 (3%)		0 (0%)	2 (2%)	1 (0%)	
Primary TU UICC Stage				0.116				0.885
I	2 (5%)	3 (5%)	4 (6%)		4 (4%)	2 (2%)	2 (3%)	
II	5 (12%)	8 (14%)	4 (6%)		9 (9%)	5 (6%)	5 (8%)	
III	19 (45%)	12 (21%)	20 (28%)		23 (22%)	17 (20%)	9 (15%)	
IV	16 (38%)	33 (59%)	44 (61%)		68 (65%)	62 (72%)	44 (73%)	
Timing of mCRC: synchronous	18 (43%)	35 (63%)	45 (63%)	0.082	75 (72%)	61 (71%)	46 (77%)	0.730
CEA at stage IV: Mean—ng/mL (SD)	128.4 (680)	116.3 (398)	98.0 (331)	0.946 †	335.4 (988)	276.3 (714)	461.0 (1404)	0.585 †
Initial metastatic site								
Hepatic	31 (74%)	40 (71%)	46 (64%)	0.477	77 (74%)	64 (74%)	50 (83%)	0.348
Pulmonary	9 (21%)	20 (36%)	23 (32%)	0.299	26 (25%)	27 (31%)	27 (45%)	0.030
Hepatic + Pulmonary	1 (2%)	4 (7%)	5 (7%)	0.650	18 (17%)	18 (21%)	21 (36%)	0.025
Peritoneal	0 (0%)	2 (4%)	10 (14%)	0.009	29 (28%)	26 (30%)	17 (28%)	0.935
Distant lymph nodes	4 (10%)	2 (4%)	3 (4%)	0.461	15 (14%)	10 (12%)	3 (5%)	0.173
Others	1 (2%)	2 (4%)	5 (7%)	0.593	13 (13%)	10 (12%)	8 (13%)	0.969

* χ² or Fisher’s exact test, except †: ANOVA. ASA: American Society of Anaesthesiologists; BMI: Body mass index; CEA: carcinoembryogenic antigen; dx: diagnosis; mCRC: metastatic colorectal cancer; SD: standard deviation; TU: tumour; UICC: Union internationale contre le cancer.

**Table 2:** Differences in the characteristics of curative (CIS) and palliative (PAT) patients.

Variable	Curative (CIS; *n* = 170)	Palliative (PAT; *n* = 250)	*p* *
Patient-related factors			
Sex: male	108 (63%)	154 (62%)	0.689
Age at first diagnosis of metastases: mean (SD)	64.7 (10.5)	69.8 (11.7)	<0.001 §
≥70 years	58 (34%)	131 (52%)	<0.001
ASA Score			<0.001
I	15 (9%)	19 (8%)	
II	106 (62%)	100 (40%)	
III	47 (28%)	108 (43%)	
IV/V	2 (1%)	23 (9%)	
BMI: mean (SD)	25.3 (4.1)	25.4 (4.5)	0.914 §
Disease/Tumour-related factors			
Primary tumour			
T stage			<0.001
T1 & T2	20 (12%)	18 (7%)	
T3	113 (67%)	123 (49%)	
T4 & Tx	37 (22%)	109 (44%)	
Nodal status			<0.001
N negative	46 (27%)	43 (17%)	
N positive	122 (72%)	181 (73%)	
Nx (unknown)	2 (1%)	26 (10%)	
R-Status (missing = 8)			<0.001
R0	152 (91%)	88 (36%)	
R1	8 (5%)	11 (5%)	
R2 or no resection	8 (5%)	145 (60%)	
Tumour differentiation (missing = 12)			0.090
G1	2 (1%)	3 (1%)	
G2	116 (71%)	147 (60%)	
G3	46 (28%)	91 (37%)	
G4	0 (0%)	3 (1%)	
Tumour sidedness			0.002
Right Colon	38 (22%)	91 (36%)	
Left Colon incl. Rectum	132 (78%)	159 (64%)	
Histology			0.745
Adeno-Carcinoma	167 (98%)	244 (98%)	
Mucinous or Signet-Cell	3 (2%)	6 (2%)	
Metastases			
Occurrence of metastases			0.001
Synchronous (≤6 months)	98 (58%)	182 (73%)	
Metachronous (>6 months)	72 (42%)	68 (27%)	
CEA at diagnosis of metastases (missing = 39)			<0.001
≤200 ng/mL	138 (93%)	174 (75%)	
>200 ng/mL	10 (7%)	59 (25%)	
Metastatic extent			<0.001
Liver limited	98 (58%)	90 (36%)	
Lung limited	39 (23%)	18 (7%)	
Liver-lung limited	11 (7%)	31 (12%)	
Other organs involvement	22 (13%)	111 (44%)	
Treatment-related factors			
CTX received at any time since mets-dx (missing = 3)	151 (89%)	190 (77%)	<0.001
Type of CTX (missing = 11)			0.006
Including IRI and/or OX	139 (84%)	67 (28%)	
5-FU-based only or no CTX (BSC)	26 (16%)	177 (73%)	
Received biological anytime since mets-dx (missing = 10)	105 (63%)	144 (59%)	0.389
Time period of diagnosis of metastases			<0.001
2003–2006	42 (25%)	104 (42%)	
2007–2010	56 (33%)	86 (34%)	
2011–2014	72 (42%)	60 (24%)	

* χ² or Fisher’s exact test, except § Mann-Whitney-U; percentage may exceed 100% due to rounding. ASA = American Society of Anaesthesiologists; BMI = Body mass index; BSC = best supportive care; CEA = carcinoembryogenic antigen; CTX = chemotherapy; IRI = irinotecan; mets-dx = diagnosis of metastases; OX = oxaliplatin; SD = standard deviation.

**Table 3:** Data on (**A**) surgical procedures and (**B**) chemotherapy for curative (CIS) patients’ group (*n* = 170).

Variable	2003–2006	2007–2010	2011–2014	*p* *
Curative patients (% of total)	42 (29%)	56 (39%)	72 (55%)	<0.001
(**A**) Surgical data				
Liver resections (% of total)	31 (21%)	40 (28%)	46 (35%)	0.041
Liver first concept (% of LR)	0 (0%)	5 (13%)	6 (13%)	0.122
Major resections (% of LR)	4 (13%)	8 (20%)	17 (37%)	0.039
Laparoscopic resection (% of LR)	0 (0%)	3 (8%)	6 (13%)	0.109
Liver metastases				
Involvement: bilobar (% of LR)	6 (19%)	10 (25%)	24 (52%)	0.004
Number: mean (range)	2.2 (1–8)	2.5 (1–9)	5.5 (1–40)	0.005
Diameter of largest lesion (cm): mean (range)	2.9 (0.9–5.7)	2.6 (0.4–7.0)	3.0 (0.4–16.0)	0.807 †
Surgical outcome LR				
Length of stay (days): mean (range)	11.2 (3–34)	14.7 (2–126)	12.4 (4–50)	0.514 †
Complications (% of LR)	9 (29%)	9 (23%)	19 (41%)	0.024
Mild (Clavien-Dindo 1-3a)	4 (13%)	4 (10%)	17 (37%)	
Severe (Clavien-Dindo 3b-4b)	5 (16%)	5 (13%)	2 (4%)	
Mortality (In-hospital)	0 (0%)	0 (0%)	1 (2%)	0.459
Lung resections (% of total)	9 (6%)	20 (14%)	23 (17%)	0.013
Thoracoscopic resection (VATS) (% of lung resections)	4 (44%)	4 (20%)	10 (44%)	0.215
Lung metastases				
Involvement: bilateral (% of lung resections)	0 (0%)	3 (15%)	5 (22%)	0.308
Number: mean (range)	1.2 (1–2)	2.5 (1–12)	2.3 (1–8)	0.359 †
Diameter of largest lesion (cm): mean (range)	1.9 (0.5–3.1)	1.8 (0.4–5.0)	1.4 (0.3–6.0)	0.496 †
Surgical outcome lung resections				
Length of stay (days): mean (range)	7.4 (2–18)	6.1 (1–15)	6.0 (2–20)	0.719
Complications (% of lung resections)	2 (22%)	3 (15%)	6 (26%)	0.216
Mild (Clavien-Dindo 1-3a)	1 (11%)	1 (5%)	6 (26%)	
Severe (Clavien-Dindo 3b-4b)	1 (11%)	2 (10%)	0 (0%)	
Mortality (In-hospital)	0 (0%)	0 (0%)	0 (0%)	-
Peritonectomy (% of total)	0 (0%)	2 (1%)	10 (8%)	0.009
Including HIPEC (% of Peritonectomy)	0 (0%)	0 (0%)	5 (50%)	-
Other curative surgery (LA/abd. organs) (% of total)	3 (2%)	3 (2%)	6 (5%)	0.372
Further curative treatment after initial metastasectomy	18 (43%)	21 (38%)	28 (39%)	0.860
Number of further treatments: mean (range)	1.6 (1–5)	1.5 (1–4)	1.6 (1–4)	0.883 †
(**B**) Chemotherapy in curative patients				
CTX received since first diagnosis of metastases	37 (88%)	52 (93%)	62 (86%)	0.460
Number of CTX cycles (months) received: mean (range)	11.5 (0–31)	9.3 (0–23)	8.8 (0–33)	0.147 †
Pseudoneoadjuvant before metastasectomy	16 (38%)	21 (38%)	27 (38%)	0.998
Adjuvant/palliative after metastasectomy	33 (79%)	44 (79%)	59 (82%)	0.694
Type of CTX scheme				
5-FU-Mono-based	11 (29%)	12 (22%)	21 (29%)	0.609
Oxaliplatin/Irinotecan-based dual therapy **				0.733
One agent	13 (34%)	23 (41%)	34 (47%)	
Both agents (sequentially)	18 (47%)	22 (39%)	27 (38%)	
Oxaliplatin/Irinotecan-based triple (FOLFOXIRI)	0 (0%)	2 (4%)	3 (4%)	0.456
Biological included ***	21 (55%)	38 (68%)	46 (64%)	0.457
Other agents ****	0 (0%)	2 (4%)	11 (15%)	0.007

* χ² or Fisher’s exact test, except †: ANOVA; ** CAPOX (Capecitabine/Oxaliplatin) or FOLFOX (Folinic acid/5-FU/Oxaliplation) or XELIRI (Capecitabine/Irininotecan) or FOLFIRI (Folinic acid/5-FU/Irinotecan); CTX=chemotherapy; *** Bevacizumab or Panitumumab, Cetuximab, Tivozanib, Matuzumab; **** Aflibercept or Regorafenib, Mitomycin C, TAS 102/Lonsurf, Phase I study agents; 5-FU = 5-Fluorouracil; abd. organs = other abdominal organs; FOLFOXIRI = Folinic acid, 5-FU, Oxaliplatin and Irinotecan; LA = lymphadenectomy of distant lymph nodes; LR = liver resections; VATS = video assisted thoracoscopic surgery.

**Table 4:** Univariable and multivariable analysis of factors associated with overall survival (whole cohort; *n* = 420).

Variable	No. (%)	Median OS (mo; 95%CI)	Univariable Analysis	*p*	Multivariable Analysis	*p*
			HR (95%CI)		HR (95%CI)	
Patient-related factors						
Sex						
Female	158 (38%)	24.4 (16.6–32.2)				
Male	262 (62%)	25.3 (21.2–29.4)	1.01 (0.81–1.26)	0.913		
Age at first mets-dx						
<70	231 (55%)	33.4 (27.3–39.5)				
≥70	189 (45%)	18.3 (14.0–22.6)	1.46 (1.18–1.81)	0.001	0.99 (0.78–1.27)	0.958
ASA Score						
I	34 (8%)	23.6 (12.9–34.3)				
II	206 (49%)	36.5 (31.0–42.0)	0.97 (0.62–1.50)	0.888	1.14 (0.72–1.82)	0.579
III	155 (37%)	17.0 (14.4–19.6)	1.63 (1.05–2.54)	0.031	1.44 (0.89–2.33)	0.134
IV/V	25 (6%)	4.5 (2.1–7.0)	6.05 (3.40–10.77)	<0.001	3.50 (1.85–6.61)	<0.001
BMI (missing = 11)						
<25.4 (=Median)	217 (52%)	22.6 (17.7–27.5)				
≥25.4	192 (46%)	28.1 (23.7–32.5)	0.90 (0.72–1.12)	0.342		
Disease/Tumour-related factors						
Primary tumour						
T stage						
T1 & T2	38 (9%)	30.5 (16.9–44.1)				
T3	236 (56%)	31.1 (26.8–35.4)	1.03 (0.70–1.53)	0.868	0.94 (0.61–1.44)	0.759
T4 & Tx	146 (35%)	15.6 (12.6–18.6)	1.69 (1.13–2.53)	0.011	1.00 (0.64–1.57)	0.996
Nodal status						
N negative	89 (21%)	41.0 (27.8–54.3)				
N positive	303 (72%)	23.2 (19.0–27.4)	1.55 (1.18–2.04)	0.002	1.52 (1.13–2.04)	0.006
Nx (unknown)	28 (7%)	12.8 (3.6–22.0)	2.91 (1.85–4.58)	<0.001	1.36 (0.81–2.26)	0.245
R-Status						
R0	240 (58%)	39.1 (34.5–43.7)				
R1	19 (5%)	12.9 (9.4–16.42)	1.94 (1.14–3.30)	0.014	1.13 (0.63–2.03)	0.686
R2/no resection/Rx	161 (38%)	15.1 (12.0–18.2)	2.84 (2.27–3.58)	<0.001	1.05 (0.75–1.46)	0.782
Tumour differentiation						
G1	5 (1%)	20.6 (3.9–37.4)				
G2	263 (63%)	30.5 (26.6–34.4)	0.77 (0.32–1.87)	0.564		
G3	137 (33%)	16.2 (10.5–21.9)	1.04 (0.42–2.54)	0.940		
G4	3 (1%)	3.7 (10.8–25.2)	1.43 (0.34–5.99)	0.626		
Gx	12 (3%)	32.5 (0.0–66.1)	0.71 (0.24–2.11)	0.535		
Tumour location (sidedness)						
Left Colon incl. Rectum	291 (69%)	29.6 (24.8–34.4)				
Right Colon	129 (31%)	15.3 (11.8–18.8)	1.59 (1.26–1.99)	<0.001	1.33 (1.04–1.69)	0.021
Histology primary tumour						
Adeno-Carcinoma	411 (98%)	25.9 (22.0–29.8)				
Mucinous or Signet-Cell	9 (2%)	12.4 (0.0–27.6)	2.80 (1.44–5.46)	0.002	3.18 (1.55–6.55)	0.002
Metastases						
Occurrence of metastases						
Metachronous (>6 months)	140 (33%)	32.5 (24.7–40.3)				
Synchronous (≤6 months)	280 (67%)	21.7 (17.7–25.7)	1.52 (1.20–1.93)	<0.001	1.25 (0.90–1.72)	0.180
CEA at mets-dx						
≤200 ng/mL	312 (74%)	28.3 (23.4–33.2)				
>200 ng/mL	69 (16%)	13.6 (9.0–18.3)	2.22 (1.68–2.93)	<0.001	1.74 (1.27–2.39)	<0.001
Missing	39 (9%)	31.7 (16.2–47.2)	0.83 (0.56–1.24)	0.833	0.94 (0.61–1.45)	0.779
Metastatic extent						
Liver limited	188 (45%)	29.8 (24.1–35.6)				
Lung limited	57 (13%)	46.8 (34.7–59.0)	0.57 (0.40–0.83)	0.003	0.70 (0.47–1.04)	0.080
Liver-lung limited	42 (10%)	25.5 (9.9–41.1)	1.46 (1.01–2.11)	0.042	0.95 (0.63–1.43)	0.803
Other organs involvement	133 (32%)	15.6 (11.9–19.3)	1.80 (1.41–2.30)	<0.001	1.24 (0.91–1.69)	0.167
Treatment-related factors						
Initial treatment strategy						
Curative	170 (41%)	52.1 (39.9–54.3)				
Palliative	250 (60%)	14.0 (11.6–16.4)	4.36 (3.42–5.57)	<0.001	3.68 (2.64–5.12)	<0.001
CTX since mets-dx (miss = 3)						
Any chemotherapy	341 (82%)	29.8 (25.7–40.0)				
BSC/no chemo	76 (18%)	4.0 (2.7–5.3)	2.42 (1.84–3.18)	<0.001	**	
Type of CTX						
Including IRI and/or OX	316 (75%)	30.8 (27.1–34.5)				
5-FU-based only/BSC/no CTX	93 (22%)	6.7 (3.3–10.1)	2.00 (1.55–2.59)	<0.001	2.57 (1.92–3.45)	<0.001
Unknown	11 (3%)	21.6 (17.2–26.0)	1.17 (0.62–2.20)	0.627	2.05 (1.06–3.99)	0.034
Received biological						
At any time since mets-dx	249 (59%)	29.8 (26.4–33.2)				
Never	161 (38%)	14.4 (8.3–20.5)	1.13 (0.90–1.42)	0.289		
Missing	10 (3%)	20.6 (16.3–24.9)	1.04 (0.53–2.03)	0.905		
Time period of mets-dx						
2003–2006	146 (35%)	21.9 (17.3–26.5)				
2007–2010	142 (34%)	24.2 (17.5–30.9)	0.91 (0.71–1.16)	0.428		
2011–2014	132 (31%)	36.5 (26.6–46.4)	0.67 (0.51–0.89)	0.005	**	

The first category serves as reference (HR 1.0); ** After sensitivity analysis, these factors were not included in the multivariable model due to (multi-)collinearity with other factors (e.g., age). ASA = American Society of Anaesthesiologists; BMI = Body mass index; BSC = best supportive care; CEA = carcinoembryogenic antigen; CTX = chemotherapy; IRI = irinotecan; mets-dx = diagnosis of metastases; miss = patients with missing values; OX = oxaliplatin.

**Table 5:** Factors associated with palliative treatment decision (PAT; logistic regression).

Variable	OR (95% CI)	*p*
**Patient factors**		
Sex: male	0.95 (0.55–1.65)	0.859
Age at diagnosis of metastases ≥70	2.22 (1.28–3.83)	0.004
ASA ≥ 3	1.39 (0.79–2.42)	0.250
BMI ≥ 25.4	1.30 (0.77–2.22)	0.326
**Tumour factors**		
Nodal positive primary tumour	1.13 (0.61–2.09)	0.697
Grading ≥ G3	0.98 (0.55–1.74)	0.939
Right-sided primary tumour	0.59 (0.33–1.07)	0.083
Synchronous metastases	2.43 (1.33–4.45)	0.004
CEA >200 ng/mL	2.86 (1.26–6.47)	0.012
Metastatic extent		
Liver limited	1 (ref)	
Lung limited	1.38 (0.60–3.18)	0.453
Combined lung & liver or other organ involvement	6.73 (3.55–12.76)	<0.001
**Time period of diagnosis of metastases**		
2003–2006	1 (ref)	
2007–2010	0.54 (0.29–1.00)	0.050
2011–2014	0.15 (0.08–0.30)	<0.001

Cases with Nx or Gx (mostly palliative patients) and with unknown CEA were excluded from logistic regression analysis. ASA = American Society of Anaesthesiologists; BMI = Body mass index; CEA = carcinoembryogenic antigen; OR = odds ratio; 95% CI = 95% confidence interval.

## Supplementary Materials

The following are available online at http://www.mdpi.com/2072-6694/11/2/218/s1: Table S1: Demographic: clinical and pathological characteristics of all patients, Table S2: Data on chemotherapy for (A) all patients (B) palliative patients (PAT) group, Table S3: Excel sheet with anonymized patient data used for this study.
