# Supplementary material for: Progressive Oncological Surgery Is Associated with Increased Curative Resection Rates and Improved Survival in Metastatic Colorectal Cancer
Source: Cancers (Basel). 2019 Feb 14;11(2):218. doi: 10.3390/cancers11020218 (PMC6406820; doi:10.3390/cancers11020218)
Supplement: Supplementary file 1 [file cancers-11-00218-s001.zip › cancers-433977-suppl-final/cancers-433977-suppl-final-checked.pdf]

Supplementary Materials: Progressive Oncological Surgery Is Associated with Increased Curative Resection Rates and Improved Survival in Metastatic Colorectal Cancer

Florian Primavesi, Stefan Stättner, Tarkan Jäger, Georg Göbel, Jaroslav Presl, Katerina Tomanová, Selina Buchner, Manuel Maglione, Thomas Resch, Jörg Hutter, Dietmar Öfner and Adam Dinnewitzer

Table S1. Demographic, clinical and pathological characteristics of all patients.

Variable	2003–2006 *n* = 146	2007–2010 *n* = 142	2011–2014 *n* = 132	*p*-value *
**Age at stage IV diagnosis**: Mean (SD)	66.7 (11.1)	68.2 (11.5)	68.2 (12.1)	0.403†
**BMI:** Mean (SD)	25.5 (4.2)	25.5 (4.2)	25.0 (4.6)	0.550†
**Sex:** Male	100 (69%)	84 (59%)	78 (59%)	0.168
**ASA**				0.530
- 1	13 (9%)	15 (11%)	6 (5%)	
- 2	71 (49.0%)	65 (46%)	70 (53%)	
- 3	52 (36%)	55 (39%)	47 (36%)	
- 4	9 (6%)	6 (4%)	9 (7%)	
- 5	0 (0%)	1 (1%)	0 (0%)	
**Primary TU Location**				0.677
- Colon	86 (59%)	89 (63%)	84 (64%)	
- Rectum	59 (40%)	51 (36%)	45 (34%)	
- Both	1 (1%)	2 (1%)	3 (2%)	
**Primary TU UICC Stage**				0.603
- I	6 (4%)	5 (4%)	6 (5%)	
- II	14 (10%)	13 (9%)	9 (7%)	
- III	42 (29%)	29 (20%)	29 (22%)	
- IV	84 (58%)	95 (67%)	88 (67%)	
**Timing of metastasis:** synchronous (6 months)	93 (64%)	96 (68%)	91 (69%)	0.624
**CEA at stage IV** (mean; ng/ml)	277.6 (915)	218.6 (622)	267.6 (1002)	0.846†
**Initial metastatic site**				
- Hepatic	108 (74%)	104 (73%)	96 (73%)	0.972
- Pulmonary	35 (24%)	47 (33%)	50 (38%)	0.039
- Hepatic + Pulmonary	19 (13%)	22 (16%)	26 (20%)	0.295
- Peritoneal	29 (20%)	28 (20%)	27 (21%)	0.987
- Distant lymph nodes	19 (13%)	12 (9%)	6 (5%)	0.046
- Others	14 (10%)	12 (9%)	13 (10%)	0.913

* χ^2 or Fisher's exact test, except †ANOVA. ASA = American Society of Anaesthesiologists; BMI = Body mass index; CEA = carcinoembryogenic antigen; SD = standard deviation.

Table S2. Data on chemotherapy for A) all patients B) palliative patients (PAT) group.

Variable	2003–2006	2007–2010	2011–2014	*p*-value *
**(A) Chemotherapy in whole cohort (% of all patients)**	***n* = 146**	***n* = 142**	***n* = 132**	
Chemotherapy received since first diagnosis of metastasis	113 (79%)	120 (85%)	108 (82%)	0.487
- Number of CTX cycles (months) received: mean (range)	10.0 (0–31)	9.5 (0–31)	7.9 (0–33)	0.055†
Type of CTX scheme				

- 5-FU-Mono based	26 (19%)	43 (31%)	37 (28%)	0.077
- Oxaliplatin / Irinotecan based dual (CAPOX, FOLFOX, XELIRI, FOLFIRI)	34 (25%)	39 (28%)	29 (22%)	0.040
- None	31 (23%)	45 (32%)	52 (39%)	
- One agent	71 (52%)	58 (41%)	51 (39%)	
- Both agents (sequentially)	0 (0%)	2 (1%)	3 (2%)	0.231
- Oxaliplatin / Irinotecan based triple (FOLFOXIRI)	73 (54%)	95 (67%)	81 (61%)	0.077
- Biological included (Bevacizumab, Panitumumab, Cetuximab, Tivozanib, Matuzumab)	0 (0%)	9 (6%)	27 (21%)	<0.001
- Other agents (Aflibercept, Regorafenib, Mitomycin C, TAS - 102/Lonsurf, Phase I study agents)				
**(B) Chemotherapy in palliative patients (% of PAT Patients)**	***n* = 104**	***n* = 86**	***n* = 60**	***p*-value**
Chemotherapy received since first diagnosis of metastasis *(missing n=2)*	76 (75%)	68 (79%)	46 (77%)	0.763
- Number of CTX cycles (months) received: mean (range)	9.5 (0–28)	9.6 (0–31)	6.7 (0–30)	0.071†
Type of CTX scheme				
- 5-FU-Mono based	15 (15%)	31 (36%)	16 (27%)	0.005
- Oxaliplatin / Irinotecan based dual (CAPOX, FOLFOX, XELIRI, FOLFIRI)	27 (28%)	28 (33%)	18 (30%)	0.303
- None	18 (18%)	22 (26%)	18 (30%)	
- One agent	53 (54%)	36 (42%)	24 (40%)	
- Both agents (sequentially)	0 (0.0%)	0 (0%)	0 (0%)	-
- Oxaliplatin / Irinotecan based triple (FOLFOXIRI)	52 (53%)	57 (66%)	35 (58%)	0.190
- Biological included (Bevacizumab, Panitumumab, Cetuximab, Tivozanib, Matuzumab)	0 (0%)	7 (8%)	16 (27%)	<0.001
- Other agents (Aflibercept, Regorafenib, Mitomycin C, TAS 102/Lonsurf, Phase I study agents)				

*χ^2 or Fisher's exact test, except †ANOVA; CAPOX = Capecitabine / Oxaliplatin; CTX=chemotherapy; 5-FU = 5-Fluorouracil; FOLFOX = Folinic acid / 5-FU / Oxaliplation; FOLFIRI = Folinic acid / 5-FU / Irinotecan; FOLFOXIRI = Folinic acid, 5-FU, Oxaliplatin and Irinotecan; LA = lymphadenectomy of distant lymph nodes; abd. organs = other abdominal organs; VATS = video assisted thoracoscopic surgery; XELIRI = Capecitabine / Irininotecan. .

Table S3. Excel sheet with anonymized patient data used for this study. (See attached file).

© 2019 by the authors. Licensee MDPI, Basel, Switzerland. This article is an open access article distributed under the terms and conditions of the Creative Commons Attribution (CC BY) license (http://creativecommons.org/licenses/by/4.0/).
